# Proteomic Differences between Tellurite-Sensitive and Tellurite–Resistant *E.coli*

**DOI:** 10.1371/journal.pone.0078010

**Published:** 2013-11-11

**Authors:** Jana Aradská, Roman Šmidák, Lenka Turkovičová, Ján Turňa, Gert Lubec

**Affiliations:** 1 Department of Molecular Biology, Faculty of Natural Science, Comenius University, Bratislava, Slovakia; 2 Department of Pediatrics, Medical University of Vienna, Vienna, Austria; Instituto de Biotecnología, Universidad Nacional Autónoma de México, Mexico

## Abstract

Tellurite containing compounds are in use for industrial processes and increasing delivery into the environment generates specific pollution that may well result in contamination and subsequent potential adverse effects on public health. It was the aim of the current study to reveal mechanism of toxicity in tellurite-sensitive and tellurite-resistant *E. coli* at the protein level.

In this work an approach using gel-based mass spectrometrical analysis to identify a differential protein profile related to tellurite toxicity was used and the mechanism of ter operon-mediated tellurite resistance was addressed.

*E. coli BL21* was genetically manipulated for tellurite-resistance by the introduction of the resistance-conferring *ter* genes on the pLK18 plasmid. Potassium tellurite was added to cultures in order to obtain a final 3.9 micromolar concentration. Proteins from tellurite-sensitive and tellurite-resistant *E. coli* were run on 2-D gel electrophoresis, spots of interest were picked, in-gel digested and subsequently analysed by nano-LC-MS/MS (ion trap). In addition, Western blotting and measurement of enzymatic activity were performed to verify the expression of certain candidate proteins.

Following exposure to tellurite, in contrast to tellurite-resistant bacteria, sensitive cells exhibited increased levels of antioxidant enzymes superoxide dismutases, catalase and oxidoreductase YqhD. Cysteine desulfurase, known to be related to tellurite toxicity as well as proteins involved in protein folding: GroEL, DnaK and EF-Tu were upregulated in sensitive cells. In resistant bacteria, several isoforms of four essential Ter proteins were observed and following tellurite treatment the abovementioned protein levels did not show any significant proteome changes as compared to the sensitive control.

The absence of general defense mechanisms against tellurite toxicity in resistant bacteria thus provides further evidence that the four proteins of the ter operon function by a specific mode of action in the mechanism of tellurite resistance probably involving protein cascades from antioxidant and protein folding pathways.

## Introduction

Tellurium (Te) is a trace element belonging to the same chemical group as selenium, sulphur and oxygen. Toxic ions are mustered mostly from industrial activities and represent a potential danger to human health. Tellurite oxyanions are highly toxic for most forms of life even at micromolar levels [1], even though the ultimate molecular mechanism underlying tellurite toxicity is not fully understood. Several different mechanisms have been proposed to account for the toxicity of tellurite. Part of it results from ROS generation as by-product of tellurite reduction [2], [3], [4], [5], either by specific superoxide dismutation by SOD or by accidental transfer of electrons to O_2_ during auto-oxidation of respiratory dehydrogenases [6]. Tellurite oxidizes cellular thiols as glutathione [7] or causes specific damage to [Fe-S] clusters present in essential enzymes [8] and may replace sulphur and/or selenium in critical metabolites or enzymes thus abating essential functions [9]. Moreover, tellurite causes lipid peroxidation with subsequent generation of toxic breakdown products like short-chain (C3–C9) aldehydes [10]. Taken together, the toxicity of tellurite results from an ability to act as a strong oxidizing agent over a variety of cell components. Most pathways that are activated in the cell after tellurite exposure tend to mitigate these effects.

Natural resistance to toxic compounds results from high adaptability of cellular systems to environmental changes. To cope with chemical stress, microorganisms use various defense mechanisms involving complementary action of distinct pathways. These include the evolution of specific mechanisms targeted against a particular dangerous agent along with the recruitment of well-established general defense [11], [12].

In the current study sensitive cells *E. coli* BL21(pACYC184) and cells carrying a tellurite resistance gene determinant from the *E. coli KL53*, one of the five genetic determinants of tellurite resistance found in gram-negative bacteria, were used. Bacteria bearing the Ter operon can grow at significantly higher concentrations of tellurite (MIC∼4 mM) in comparison to the wild type form (MIC∼4 µM). This operon was also detected within the pathogenicity island in the genome of foodborne pathogens such as *E. coli* O157:H7 [13]. Homologous genes have also been found in *Shigella flexneri*, *Yersinia pestis* [1], *Klebsiella pneumoniae* [14], *Vibrio cholerae*, *Proteus mirabilis* [15], *Deinococcus radiodurans* [13], etc. The corresponding DNA fragment conferring the resistance is comprised of four essential ORFs that were named *terB, terC, terD and terE*. The functional properties of the four gene products and therefore the mechanism of resistance mediated by *ter* operon remains elusive.

Several studies have proposed a role for *ter* genes in resistance to bacteriophages, colicins, oxidative stress which was also shown to be important for *E. coli* pathogens to overcome the host immune system [13], [16], [17]. Furthermore, Anantharaman et al. [18] recently employed a systemic approach to predict the involvement of the *ter* gene at different levels of highly complex bacterial defense systems involving sensing, signalling pathways or DNA repair. Taken together, data reported so far are anecdotal and warrant a systematic approach for the determination of probably involved pathways and cascades involved in tellurite sensitivity: It was the aim of the study to provide insight into proteomic differences between *E.coli* sensitive or resistant to tellurite by a gel-based proteomic approach using mass spectrometry.

Herein the comparative analysis of the total cell lysate proteomes from sensitive cells *E. coli* BL21(pACYC184) and resistant cells *E. coli* BL21(pLK18) against tellurite is reported and the identification of proteins from several networks involved in the molecular stress response were revealed.

## Results

### Determination of the “sub-lethal” concentration of potassium tellurite

**Figure 1:**
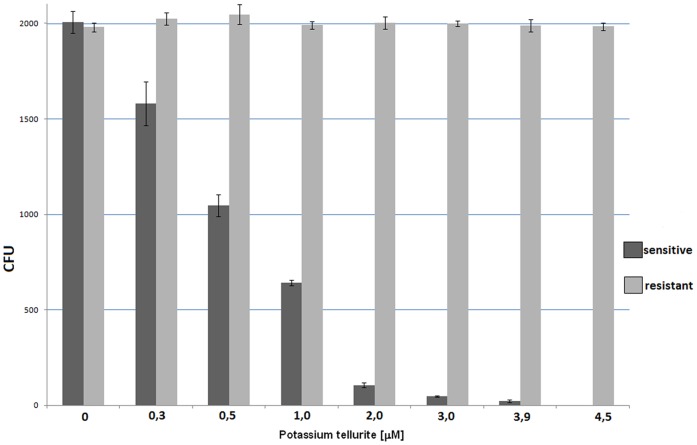
Determination of the “sub-lethal” concentration for the sensitive strain *E. coli* BL21(pACYC184). Cells were incubated for a defined time interval of 10°C in the presence of potassium tellurite (at 0.3–4.5 µM).

The minimum inhibitory concentration (MIC) of potassium tellurite was determined for the sensitive strain *E. coli* BL21(pACYC184) as 4.5 µM. After 10 min incubation in tellurite-supplemented LB medium ≈50 percent of cells survived at 0.5 µM and <1 percent of cells survived at 3.9 µM K_2_TeO_3_. A concentration representing LD_50_ (0.5 µM) and the “sub-lethal” concentration of tellurite (3.9 µM; concentrations that led to <1% survival but enabling re-culturing from surviving cells) as well as the time of incubation (10 min.) were used in all subsequent experiments. This concentration had no effect on viability of resistant cells (Fig. 1).

### Effect of tellurite toxicity on the proteomic profile of sensitive cells

**Figure 2:**
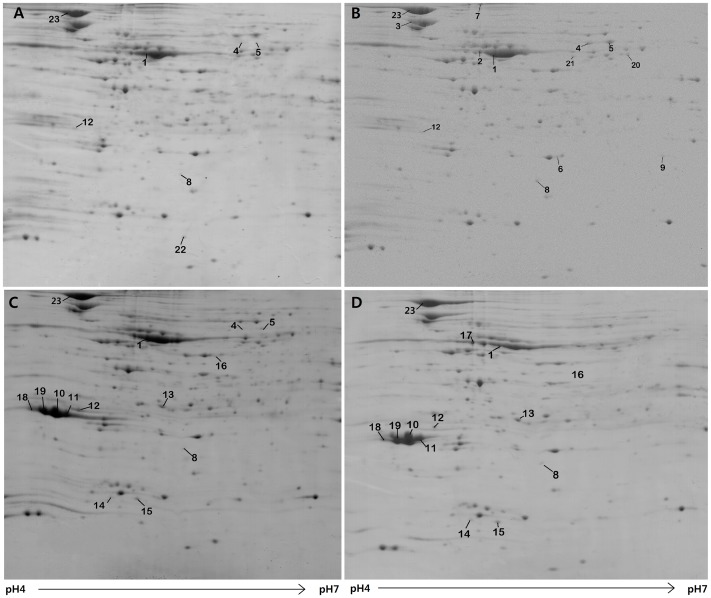
Images of two 2D electrophoresis gels. Crude protein extracts (600 µg) were separated on strips pH 4–7 and SDS-PAGE on 15% polyacrylamide gradient gels. Spots with significant differences in expression level are marked on the gel: *E. coli BL21/pACYC184* cells (A) untreated and (B) treated with K_2_TeO_3_, and *E. coli BL21/pLK18* cells (C) untreated and (D) treated with K_2_TeO_3_.

Proteins from the total lysate of *E. coli BL21*(pACYC184) grown in pure LB culture and in LB culture with “sub-lethal” concentrations of K_2_TeO_3_ were subjected to comparative proteomics analysis using 2-D gel electrophoresis. Preliminary experiments covering pH 3–10 demonstrated that nearly all proteins presented at acidic isoelectric points (pIs) below pH 7 (not shown). Therefore, proteomics analysis focused on the protein-dense area pH 4–7 (Fig. 2). Comparison of the 2-DE patterns in three replicate continuous cultures incubated with and without potassium tellurite revealed significant differences (p<0.05) in the silver blue stained gel images. From the PDQuest 2-D analysis software (BioRad, Munich, Germany) followed by one-way ANOVA, 592 spots were observed and significant changes in protein levels for 12 distinct protein spots were detected (Fig. 2 A,B) . The 12 protein spots with statistically significant differential levels between tellurite treated- and untreated groups were selected for identification by peptide mass fingerprinting using nano-LC-ESI-CID/ETD-MS/MS. Peptide mass analysis revealed that 11 proteins were up-regulated in sensitive cells treated with tellurite and one protein was down-regulated (Table 1).

**Table 1:** Proteins with at least a two-fold difference in expression (P<0.05) identified by nano-LC-ESI-CID/ETD-MS/MS for *E. coli BL21*(pACYC184) and *E. coli BL21*(pLK18).

Protein identity	Spot number	Accession number	S	S+Te	R	R+Te
Translation elongation factor Tu	1	gi/417161103		+		
	2	gi/417161103		+		
	12	gi/427806532			+	+
GroEL	3	gi/218551413		+		
Transcription termination factor Rho	4	gi/15804373		+		
Tryptophanase	5	gi/41936		+		
Phage shock protein A	6	gi/432376500		+		
Kat G	7	gi/170083412		+		
Superoxide dismutase	8	gi/15802070		+		
	9	gi/15804498		+		
Cysteine desulfurase	20	gi/432801838		+		
Hypothetical oxidoreductase YqhD	21	gi/326347283		+		
ε- Subunit of the F1-Atp synthase	22	gi/6729899	+			
DnaK	23	gi/26106333		+		+
Phosphopentomutase	17	gi/417616312				+
TerB	15	gi/15800694			+	+
	14	gi/157412083			+	+
TerC	16	gi/116743421			+	+
TerD	11	gi/157412085			+	+
	13	gi/157412085			+	+
	18	gi/157412085			+	+
	19	gi/157412085			+	+
TerE	10	gi/417268043			+	+
	11	gi/417250801			+	+
	13	gi/408604652			+	+
	19	gi/417250801			+	+

**S** - *E. coli BL21*(pACYC184) untreated, **S+Te** *E. coli BL21*(pACYC184) treated with K_2_TeO_3_, **R** - *E. coli* BL21(pLK18) untreated, **R+Te** *E. coli BL21*(pLK18) treated with K_2_TeO_3_, **+** - up-regulation of a protein in a specific strain and condition.

### Effect of tellurite toxicity on the proteomic profile of resistant cells

As shown in table 1 proteins DnaK and phosphopentomutase presented with increased levels in the tellurite-treated group. Figure 2 (C and D) shows representative 2-DE patterns for *E.coli BL21*(pLK18) without and with tellurite exposure. The corresponding mass spectra for Ter proteins are shown in Figures S1, S2, S3, S4.

### Comparison of protein expression profiles in sensitive and resistant cells in the presence of potassium tellurite

In order to establish a link between tellurite sensitivity and proteomic patterns, and to pinpoint proteins that are probably implicated in tellurite resistance, we compared the 2-DE expression pattern of the sensitive cells *E. coli BL21*(pACYC184) with the pattern of the resistant cells *E. coli BL21*(pLK18).

From the profile analysis combined with statistical tests, a total of 26 spots representing 17 individual proteins were found to show significant changes (p<0.05) when all groups were compared and these are marked in Figure 2. Amongst these proteins, 5 were clearly overexpressed in *E. coli BL21*(pLK18) cells and 11 were overexpressed in *E. coli BL21*(pACYC184) cells. Most of these spots (12 spots) showed 2-fold higher levels following tellurite treatment. The spots of interest were punched from the gels, in-gel digested and analysed by nano-LC-ESI-MS/MS (Table 1 and Table S1). Proteins were reliably identified by the mass spectrometrical technique applied using two independent mass spectrometrical principles, CID and ETD.

### Determination of the selected enzymatic activities associated with tellurite stress-induced protein expression

Proteomic profiles showed up-regulation of Fe and Mn superoxide dismutase, catalase (KatG) and oxidoreductase YqhD in sensitive cells after exposure to tellurite. To determine the corresponding enzymatic activities, catalase (CAT), superoxide dismutase (SOD) and aldehyde reductase YqhD activities were assayed in cell lysates of tellurite-sensitive and tellurite-resistant cells obtained before and after tellurite exposure at 0.5 µM and 3.9 µM. The cells were cultured as given above.

**Figure 3:**
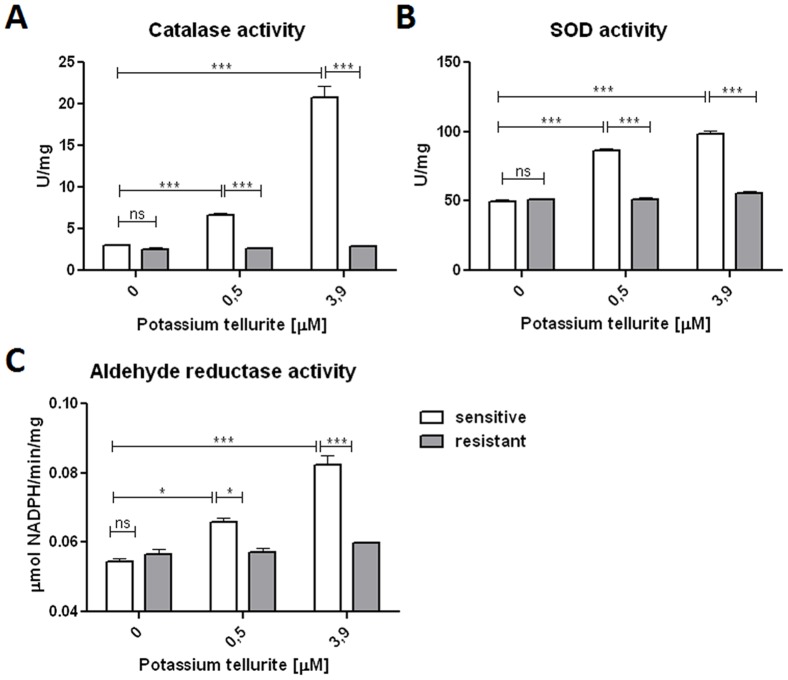
Effect of potassium tellurite on *E. coli* catalase, superoxide dismutase and aldehyde reductase activity. (**A**) Activity of catalases, (**B**) superoxide dismutases and (**C**) YqhD aldehyde-reductase activity in crude protein extracts of tellurite-sensitive and tellurite resistant *E. coli* BL21 untreated or treated with 0.5 µM and 3.9 µM of K2TeO3. Cells were collected after 10 min and catalase activity (µmol hydrogen peroxide/min/mg protein), SOD activity (U/mg protein) and YqhD activity (µmol/min/mg) were determined. Error values represent the standard deviation of triplicate experiments, ns - not significant, *P<0.05, ***P<0.001.

Crude extracts obtained from tellurite-treated sensitive cells showed a significant increase in catalase activity as compared to the basal levels exhibited by untreated sensitive cells. In contrast, catalase activity of resistant cells failed to show significant changes after tellurite treatment and was comparable to untreated controls (Fig. 3A). Similarly, total SOD activity was significantly increased in tellurite-sensitive *E. coli* cells after tellurite treatment, while extracts prepared from tellurite-resistant *E. coli* cells did not show significant changes in superoxide dismutase activity (Fig. 3B). Induction of SOD activity in sensitive cells as a result of tellurite exposure suggests that the stress conditions generated by K_2_TeO_3_ in *E. coli* may be associated, at least in part, with intracellular generation of superoxide.

The *E. coli* oxidoreductase, YqhD has a strong NADPH-dependent aldehyde reductase activity against short-chain aldehydes with weak or no apparent alcohol dehydrogenase activity. It was previously proven that YqhD eliminates the toxic aldehyde acrolein, generated during tellurite mediated stress in *E. coli* [10]. In consistence with increased density of YqhD protein spot in tellurite-treated sensitive cells on 2-DE, the corresponding cell lysates showed significantly higher NADPH-dependent aldehyde reducase activity toward acrolein (Fig. 3C). No increase in aldehyde reductase activity was detected for tellurite resistant cells following tellurite treatment. No sample showed NADP^+^-dependent dehydrogenase activity using methanol, ethanol, butanol, isopropanol as substrates (not shown).

### Western blotting

**Figure 4:**
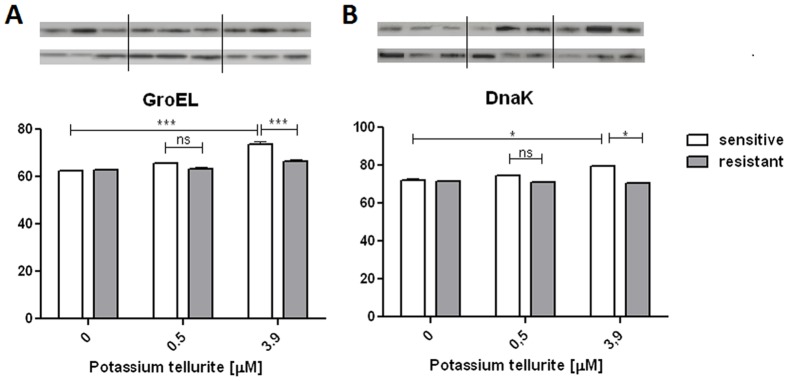
The immunoblotting patterns of proteins verifying results from two-dimensional gel electrophoresis are shown. Quantification results expressed as arbitrary units of optical density are shown (**A**) GroEL, (**B**) DnaK. Experiments were carried out in triplicates; ns - not significant, *P<0.05, ***P<0.001.

Two of differentially expressed proteins in 2-DE experiments were subjected to Western blotting analyses to confirm the differential expression. DnaK and GroEL were overexpressed in sensitive cells treated with 3.9 µM potassium tellurite (Fig. 4). This result was consistent with the observation in 2-DE analysis, showing at least two-fold higher expression level in sensitive cells than in resistant cells. Sensitive cells treated at the LD_50_ were comparable to untreated controls (Fig. 4).

## Discussion

The major outcome of the study is the identification of proteins that may be involved in the mechanism of toxicity and resistance against tellurite. Basically, in the response to tellurite-mediated stress, sensitive *E. coli* BL21 (pACYC184) cells trigger a coordinated expression of a number of proteins involved in protection against oxidative stress: These include Mn and Fe superoxide dismutases, catalase KatG and oxidoreductase YqhD. SOD, as a part of the defense system against oxidative damage in aerobic organism, catalyzes superoxide anions (O_2_^−^) to O_2_ and H_2_O_2_ that are subsequently reduced to H_2_O by the H_2_O_2_-scavenging enzyme catalase. Therefore, SOD and CAT were proposed to limit the accumulation of reactive oxygen species (ROS), by-products of tellurite reduction [19]. Extracts prepared from tellurite-treated sensitive *E. coli* cells exhibited 5-fold increased catalase activity and almost 2-fold increased superoxide dismutase activity and indeed, these observations are in agreement with previous reports [4]. The adverse effects of ROS include formation of lipid peroxidation that in turn results in generation of highly reactive toxic short chain aldehydes, such as acrolein [10]. Lee et al. showed that transcription of the yqdH gene is induced in the presence of prooxidants including potassium tellurite and YqhD was suggested to contribute to a specific defense mechanism against reactive aldehydes generated by membrane peroxidation in *E. coli* [20]. In the current study YqhD protein levels were consistently and remarkable upregulated in sensitive *E. coli* cells after tellurite treatment. Expression levels of SOD, KatG and YqhD in resistant cells treated with tellurite were equivalent to sensitive untreated cells as well as the corresponding enzymatic activities. In tellurite-sensitive cells up-regulation of cysteine desulfurase, known to be involved in repairing oxidatively damaged [Fe–S] clusters [21] as well as **t**ranscription termination factor Rho, required for oxidative stress survival [22] and tryptophanase, an enzyme catalysing the synthesis of indole were observed. Indole is a signaling molecule which suppresses growth of the bacterial biofilm and oxidative and heavy metal stresses inhibit formation of *Escherichia coli* biofilm via indole signalling [23]. Alltogether, the abovementioned findings on the antioxidant enzymes are indicating a mechanism in which tellurite toxicity results from the generation of O_2_^−^ radicals formed upon tellurite reduction.

Tellurite per se or tellurite-induced oxidative stress in sensitive cells led to increased expression of proteins mediating protein folding, protein synthesis and metabolism: GroEL, DnaK, and EF-Tu at “sub-lethal” doses. As GroEL and DnaK levels were comparable to controls at LD_50_ the significant increase of these two proteins observed at “sub-lethal” doses may be simply due to death phenomena. Apart from its role as an elongation factor, EF-Tu serves a chaperone-like function in protein folding by providing protection against thermal stress [24]. Increased expression of elongation factor EF-Tu during tellurite-mediated oxidative challenge in both, sensitive and resistant cells after exposure to tellurite was demonstrated and three distinct forms of EF-Tu (spots 1, 2, 12) were specifically expressed. Up-regulated GroEL and DnaK, known to be regulated by oxidative stress [25], [26] revealed increased expression in tellurite-treated sensitive cells although it remains open whether this finding may represent a mechanism in tellurite sensitivity. In addition to protein handling cascades given above transcription termination factor Rho was differentially expressed following tellurite-treatment in sensitive cells: this protein shows multiple functions as a multitask housekeeper and gene regulator [27] and Gao and coworkers have shown that exposure of *E.coli* to mercury leads to overexpression of transcription termination factor Rho [28]. The question, if transition metals in general are leading to increased levels of transcription termination factor Rho as a probable defense mechanism remains open but should be addressed in a follow up study.

Sensitive cells grown in the presence of tellurite showed higher levels of phage-shock protein A (PspA). The peripheral membrane protein PspA is induced under stressfull conditions and is assumed to assist in the maintenance of the membrane potential. PspA is able to suppress proton leakage of the membranes and PspA is suppressing proton leakage of damaged liposomes made from *E. coli* total lipids [29]. Although speculative, PspA may be induced following possible membrane defects generated by tellurite.

Decreased level of the epsilon subunit of the F1-ATP synthase was observed. ATP synthase is the universal enzyme that manufactures ATP from ADP and phosphate by using the energy derived from a transmembrane proton gradient [30]. Previous work showed that in the sensitive strain the transmembrane pH gradient was dissipated and intracellular ATP levels were rapidly depleted upon exposure to tellurite. No differences could be observed in intracellular ATP levels, the presence or absence of a transmembrane pH gradient or the levels of phosphorylated glycolytic intermediates when resistant cells were studied by ^31^P NMR in the presence or absence of tellurite [31].

In the resistant *E. coli* cells bearing plasmid pLK18 all four essential Ter proteins were identified. Plasmid pLK18 originates in the low-copy-number plasmid pACYC184 and carries the *terB*, *terC*, *terD* and *terE* genes [32]. Two spots (number 14, 15) were representing TerB, one spot was shown for TerC, four different spots have been identified as TerD (spots 11, 13, 18, 19) and four spots as TerE (spots 10, 11, 13, 19) that probably corresponds to different isoforms of these proteins or proteins with post-translational modifications.

Increased antioxidant protein levels were observed in *sensitive* cells in the presence of tellurite but not in the resistant counterpart treated with tellurite. This provides further evidence for different mechanisms to cope with tellurite-mediated oxidative stress in sensitive and resistant cells. The finding of increased phosphopentomutase levels in tellurite-treated resistant cells may represent activation of carbohydrate metabolism although the biological meaning remains elusive.

In untreated sensitive and resistant cells only EF-Tu and ATP synthase were presenting with differential expression probably reflecting tellurite sensitivity.

### Conclusion

Taken together, the antioxidant defense including probably repair of oxidative damage by cysteine desulfurase was induced by tellurite treatment in terms of SOD, catalase, oxidoreductase YqhD in sensitive cells at LD_50_ and “sub-lethal” doses exclusively. In addition, heat shock and chaperone proteins were induced by tellurite only in sensitive cells at “sub-lethal” doses. Changes of protein handling, i.e. protein synthesis and chaperoning following were addressed as tentative mediators of tellurite sensitivity or representing toxicity and cell death at the “sub-lethal” dose. In resistant cells major differences between tellurite treated and untreated cells at the protein level were overexpression of DnaK in tellurite treated cells rather than antioxidant defence mechanisms. DnaK is a major factor in the stress response in bacteria and may also be implicated in toxicity and the resistance against tellurite in both, sensitive and resistant cells, while tellurite induced the stress-related protein GroEL only in sensitive bacteria at highly toxic doses. The presence of Ter genes with several expression forms was shown at the protein level and it remains to be shown whether Ter genes or its downstream mediators are responsible for conferring resistance to tellurite. It is intriguing that tellurite-resistant cells seem to fail generating defense mechanisms at the protein level but a possible interpretation is that such defense mechanisms may be expected to occur at the thousandfold higher dose than that administered in order to compare the two cell lines.

## Materials and Methods

### Bacterial strains and growth conditions

A tellurite-sensitive strain *E. coli* BL21 bearing the plasmid pACYC184 (*E. coli* BL21/pACYC184) and a resistant strain *E. coli* BL21 carrying plasmid pLK18 [4] (*E. coliBL21*/pLK18) were used in all experiments. Cells were routinely grown in LB medium with chloramphenicol at 37°C with shaking

### Determination of minimal inhibition concentration

Cells were grown overnight and diluted one hundred-fold with fresh LB medium. When the OD600 was ∼0.5, potassium tellurite (Sigma, Prague, CZ) was added to obtain concentrations of 0.3, 0.5, 1.0, 2.0, 3.0, 3.9 and 4.5 µM at 10 min of incubation. Aliquots were diluted 10^6^-fold and plated in solid LB. After incubating overnight at 37°C, the number of colony forming units (CFU) was determined. MIC was defined as the lowest concentration that prevented the development of visible growth. LD_50_ was determined as 0.5 µM. For proteomic analysis using 2-DE followed by mass spectrometrical identification of proteins the “sub-lethal” concentration of 3.9 µM was used. Following identification of proteins with aberrant levels by tellurite toxicity at the “sub-lethal” concentration, activities of these proteins (superoxide dismutase, catalase and aldehyde-reductase) were also determined using a concentration at the LD_50_ in order to rule out differences in protein levels simply due to death effects.

### Preparation of protein extract for 2-DE

Cells of *E. coli BL21* (pACYC184) and *E. coli BL21* (pLK18) were grown in LB medium with chloramphenicol. When the OD600 was ∼0.5, cells were incubated without or with potassium tellurite (3.9 µM) for 10 min. Cells were harvested by centrifugation (10,000×*g*, 15 min, 4°C). The pellets were washed with 20 mM TrisHCl, pH 7.5 and directly resuspended in 20 mM TrisHCl, pH 7.5, 6 M urea, 2 M thiourea, 4% CHAPS, 1 mM EDTA, 1 mM PMSF and cells were disrupted by sonication. Cell debris was removed by centrifugation (10,000×*g*, 1 h, 4°C) to yield the total protein lysate. The protein concentrations of samples were determined using the BCA protein assay kit (Pierce, Rockford, USA).

### 2-DE

Samples were diluted in IEF buffer containing 8 M urea, 2 M thiourea, 4% CHAPS, 40 mM Tris base, 0.5% (IPGphor) Carrier (Resolyt 3–10) ampholytes and 0.008% bromophenol blue, to yield the desired protein amount (600 µg). The diluted samples were used to rehydrate 18 cm IPG strips (linear gradient pH 3–10 and 4–7; Bio-Rad, Munich, Germany) at 20°C for 16 h. IEF was performed using a horizontal electrophoretic apparatus (IPGphore, GE Healthcare, Uppsala, Sweden). The isoelectric focusing started at 200 V and the voltage was gradually increased to 8,000 V at 4 V/min and kept constant for a further 3 h (150,000 Vh total). Before the second dimension, strips were equilibrated twice for 15 min with gentle shaking in 10 mL SDS equilibration buffer (50 mM TrisHCl, pH 8.8, 6 M urea, 30% v/v glycerol, 2% w/v SDS, 0.008% bromophenol blue). DTT (1% w/v) was added at the first incubation for 15 min and 4% (w/v) iodoacetamide instead of DTT at the second incubation step for 15 min. The second dimension separation was performed using a 15% polyacrylamide gel of 1 mm thickness.

Gels were run at 50 V for 1.5 h and at 200 V until the tracking dye had reached 0.5 cm from the bottom of the gel. After protein fixation for 2 h in 50% methanol and 5% acetic acid, gels were stained using blue silver [33] for 16 h and the excess of dye was removed from the gels with distilled water.

### Image analysis of 2-DE gels and tryptic digestion

Gels were scanned on a Image Scanner (Amersham Pharmacia Biotech, Piscataway, NJ). Image analysis was performed using the PDQuest software (Bio-Rad, Munich, Germany). Three gels were produced from independent cultures for each strain and each condition and spots present on the three gels were used for comparison. Spot volume intensities were normalized to the total intensity of valid spots. Qualitative and quantitative analyses were performed. Proteins were considered differentially produced when spot intensities passed the threshold at the level of P<0.05. Selected spots were manually excised from the gels and placed in a 0.5 mL protein lobind tube (Eppendorf, Hamburg, Germany). Gel pieces were washed with 10 mM ammonium bicarbonate for 30 min and then with 10 mM ammonium bicarbonate, 50% acetonitrile for 30 min. The steps above were repeated until the color disappeared. 100 µL 100% acetonitrile was added to each tube and incubated for 10 min. Gel pieces were dried completely in a Speedvac Concentrator (Eppendorf, Hamburg, Germany). Dried gel pieces were rehydrated and digested *in situ* with 12.5 ng/µL trypsin (Promega, Madison, USA) solution buffered in 25 mM ammonium bicarbonate. Gel pieces were incubated for 16 h (overnight) at 37°C. Supernatants were transferred to new 0.5 mL tubes, and the gel pieces extracted with 10 µL 1% formic acid/5 mM n-Octyl β-D-glucopyranoside, 10 µL 0.1% formic acid, and 10 µL 0.1% formic acid/20% acetonitrile for 30 min. Samples in the extraction buffer were pooled in a 0.5 mL tube.

### Protein Identification with nano-LC-ESI-CID/ETD-MS/MS

Peptide analysis by nano-LC-ESI-CID/ETD-MS/MS was performed as described [34]. 40 µL of the extracted peptides were analyzed by nano-LC-ESI-CID/ETD-MS/MS. The HPLC used was an Ultimate 3000 system (Dionex Corporation, Sunnyvale, CA) equipped with a PepMap100 C-18 trap column (300 µm×5 mm) and PepMap100 C-18 analytic column (75 µm×150 mm). The gradient was (A = 0.1% formic acid in water, B = 0.08% formic acid in acetonitrile) 4–30% B from 0 to 105 min, 80% B from 105 to 110 min, and 4% B from 110 to 125 min. The flow rate was 300 µL/min. An HCT ultra ETDII PTM discover system (Bruker Daltonics, Bremen, Germany) was used to record peptide spectra over the mass range *m/z* 350–1,500 and MS/MS spectra were acquired by an information-dependent data acquisition method over the mass range *m/z* 100–2,800. Repeatedly, MS spectra were recorded followed by four data-dependent CID-MS/MS spectra and four ETD-MS/MS spectra generated from the three highest intensity precursor ions. The voltage between the ion spray tip and the spray shield was set to 1,400 V. Dry nitrogen gas was heated to 150°C, and the flow rate was 10 L/min. The collision energy was set automatically according to the mass and charge state of the peptides chosen for fragmentation. Multiply-charged peptides were chosen for MS/MS experiments and peak lists were generated by Data Analysis 4.0 (Bruker Daltonics, Bremen, Germany). All searches were performed against the latest NCBIKB or protein identification using the search algorithm MASCOT. Search parameters were set as follows: enzyme selected as trypsin with 2 maximum missed cleavage sites, species limited to *Escherichia coli*, a mass tolerance of 0.2 Da for peptide tolerance, 0.5 Da for MS/MS tolerance, fixed modification of carbamidomethyl (C), and variable modification of methionine oxidation (M) and phosphorylation (STY). Positive protein identifications were based on a significant MOWSE score.

### Enzymatic activity

Cells of *E. coli BL21* (pACYC184) and *E. coli BL21* (pLK18) were grown in LB medium with chloramphenicol. When the OD600 was ∼0.5, cells were incubated without or with 0.5 µM and 3.9 µM potassium tellurite for 10 min. Cells were pelleted and resuspended in PBS pH 7.2, disrupted by sonication on ice and extracts cleared by centrifugation. Aliquots of cell-free extracts were assayed for catalase, superoxide dismutase and aldehydes reductase activity following determination of protein concentration as given above.

Catalase activity was assayed spectrophotometrically [35]. 967 µl of hydrogen peroxide (50 mM potassium phosphate pH 7.2; 16 mM H_2_O_2_) was added to 33 µL of the cell extract and the decrease in the OD_240_ was monitored. The specific activity of catalase was calculated as [1,000×(OD_240_/time of incubation)]/[43.6×(mg of protein/mL of reaction mixture)] [36].

SOD activity was measured by a commercially available colorimetric assay kit (Abcam, Cambridge, UK). This method employs xanthine oxidase to generate superoxide radicals which react with 2-(4-Iodophenyl)- 3-(4-nitrophenyl)-5-(2,4-disulfophenyl)- 2H-tetrazolium (WST-1) to form a yellow formazan dye. SOD activity is measured by the degree of inhibition of this reaction. Total protein lysates were diluted 10-fold with 10 mM phosphate buffer, pH 7.2. The assay was performed at 37°C and the plate was read after twenty minutes at 450 nm. One unit of SOD is defined as the amount of enzyme needed to exhibit 50% dismutation of the superoxide radicals.

The YqhD aldehydes reductase assay was performed as described previously [10]. Total protein extracts were assayed by observing the oxidation of NADPH to NADP+ at 340 nm. The assay mixture (1 mL) consisted of 100 mM acrolein (Sigma, Prag, CZ), 50 mM sodium phosphate buffer (pH 7.2) and 0,5 mM NADPH. 1 U of enzyme activity was defined as the amount of enzyme that oxidizes 1 pmol of NADPH per min at 37°C.

### Western blotting

Equal amounts of proteins (20 µg) were subjected to 1D-SDS-PAGE (10% separating gel) followed by electroblot to PVDF membranes (Millipore, Darmstadt, Germany) using a semi-dry Bio-Rad transfer system. Membranes were blocked in blocking-buffer (5% non-fat dry milk, TBS, 0.1% Tween-20) for 1 h at 23°C, followed by incubation with diluted primary antibody anti-DnaK (1∶10,000) and anti-GroEL (1∶2,000) (Abcam, Cambridge, UK) overnight at 4°C. Membranes were washed six times by gentle agitation in TBS containing 0.1% Tween 20. Membranes were incubated with peroxidase-coupled anti-rabbit IgG (1∶20,000; Abcam, Cambridge, UK) or anti-mouse IgG (1∶10,000; Abcam, Cambridge, UK) secondary antibodies. Membranes were developed with ECL western blotting detection system (GE Healthcare, Uppsala, Sweden).

### Data analysis

In general, data are expressed as mean ± standard deviation. Differences between experimental groups were analyzed using one-way ANOVA. *P* values less than 0.05 were considered statistically significant. All statistics analyses were performed with GraphPad Prism 5.0 software (Graphpad Software Inc., San Diego CA, USA).

## Supporting Information

Figure S1 **Representative CID MS/MS spectra of tryptic peptide KLVFAVTIYDAEARKQNFGMVSNDFMR of TerB protein.**
(TIF)

Figure S2 **Representative CID MS/MS spectra of tryptic peptide FAGFLYIHHGAELASVFVTGYALEK of TerC protein.**
(TIF)

Figure S3 **Representative CID MS/MS spectra of tryptic peptide TGEGDGDDESLK of TerD protein.**
(TIF)

Figure S4 **Representative CID MS/MS spectra of tryptic peptide KQNFGMVSNSFMR of TerE protein.**
(TIF)

Table S1 **Proteins with at least a two-fold difference in expression (P<0.05) identified by nano-LC-ESI-CID/ETD-MS/MS for** ***E. coli BL21*** **(pACYC184) and** ***E. coli BL21*** **(pLK18).**
(XLSX)

## References

[1] Taylor DE (1999) Bacterial tellurite resistance. Trends Microbiol 7: 111–115. 10203839 10.1016/s0966-842x(99)01454-7

[2] Borsetti F, Tremaroli V, Michelacci F, Borghese R, Winterstein C, et al (2005) Tellurite effects on Rhodobacter capsulatus cell viability and superoxide dismutase activity under oxidative stress conditions. Res Microbiol 156: 807–813. 15946826 10.1016/j.resmic.2005.03.011

[3] Calderón IL, Arenas FA, Pérez JM, Fuentes DE, Araya MA, et al (2006) Catalases are NAD(P)H-dependent tellurite reductases. PLoS ONE 1: 1932–6203. 10.1371/journal.pone.0000070 PMC1762332 17183702

[4] Perez JM, Calderon IL, Arenas FA, Fuentes DE, Pradenas GA, et al (2007) Bacterial toxicity of potassium tellurite: unveiling an ancient enigma. PLoS One 2: e211. 17299591 10.1371/journal.pone.0000211 PMC1784070

[5] Tremaroli V, Fedi S, Zannoni D (2007) Evidence for a tellurite-dependent generation of reactive oxygen species and absence of a tellurite-mediated adaptive response to oxidative stress in cells of *Pseudomonas pseudoalcaligenes* KF707. Arch Microbiol 187: 127–135. 17013634 10.1007/s00203-006-0179-4

[6] Messner KR, Imlay JA (2002) Mechanism of superoxide and hydrogen peroxide formation by fumarate reductase, succinate dehydrogenase, and aspartate oxidase. J Biol Chem 277: 42563–42571. 12200425 10.1074/jbc.M204958200

[7] Turner RJ, Aharonowitz Y, Weiner J, Taylor DE (2001) Glutathion is a target in tellurite toxicity and is protected by tellurite resistance determinants in *Escherichia coli* . Can J Microbiol 47: 33–40. 15049447

[8] Turner R, Weiner J, Taylor D (1999) Tellurite-mediated thiol oxidation in *Escherichia coli* . Microbiology 145: 2549–2557. 10517608 10.1099/00221287-145-9-2549

[9] Garberg P, Engman L, Tolmachev V, Lundqvist H, Gerdes R, et al (1999) Binding of tellurium to hepatocellular selenoproteins during incubation with inorganic tellurite: consequences for the activity of selenium-dependent glutathione peroxidase. Int J Biochem Cell Biol 31: 291–301. 10216961 10.1016/s1357-2725(98)00113-7

[10] Perez JM, Arenas FA, Pradenas GA, Sandoval JM, Vasquez CC (2008) Escherichia coli YqhD exhibits aldehyde reductase activity and protects from the harmful effect of lipid peroxidation-derived aldehydes. J Biol Chem 283: 7346–7353. 18211903 10.1074/jbc.M708846200

[11] Masel J, Siegal ML (2009) Robustness: mechanisms and consequences. Trends Genet 25: 395–403. 19717203 10.1016/j.tig.2009.07.005 PMC2770586

[12] Venancio TM, Balaji S, Geetha S, Aravind L (2010) Robustness and evolvability in natural chemical resistance: identification of novel systems properties, biochemical mechanisms and regulatory interactions. Mol Biosyst 6: 1475–1491. 20517567 10.1039/c002567b PMC3236069

[13] Taylor DE, Rooker M, Keelan M, Ng LK, Martin I, et al (2002) Genomic variability of O islands encoding tellurite resistance in enterohemorrhagic *Escherichia coli* O157:H7 isolates. J Bacteriol 184: 4690–4698. 12169592 10.1128/JB.184.17.4690-4698.2002 PMC135296

[14] Chen YT, Chang HY, Lai YC, Pan CC, Tsai SF, et al (2004) Sequencing and analysis of the large virulence plasmid pLVPK of *Klebsiella pneumoniae* CG43. Gene 337: 189–198. 15276215 10.1016/j.gene.2004.05.008

[15] Toptchieva A, Sisson G, Bryden LJ, Taylor DE, Hoffman PS (2003) An inducible tellurite-resistance operon in Proteus mirabilis. Microbiology 149: 1285–1295. 12724390 10.1099/mic.0.25981-0

[16] Whelan KF, Colleran E, Taylor DE (1995) Phage inhibition, colicin resistance, and tellurite resistance are encoded by a single cluster of genes on the IncHI2 plasmid R478. J Bacteriol 177: 5016–5027. 7665479 10.1128/jb.177.17.5016-5027.1995 PMC177279

[17] Whelan KF, Sherburne RK, Taylor DE (1997) Characterization of a region of the IncHI2 plasmid R478 which protects *Escherichia coli* from toxic effects specified by components of the tellurite, phage, and colicin resistance cluster. J Bacteriol 179: 63–71. 8981981 10.1128/jb.179.1.63-71.1997 PMC178662

[18] Anantharaman V, Iyer LM, Aravind L (2012) Ter-dependent stress response systems: novel pathways related to metal sensing, production of a nucleoside-like metabolite, and DNA-processing. Mol Biosyst 8: 3142–3165. 23044854 10.1039/c2mb25239b PMC4104200

[19] Yao X, Min H, Lv Z (2006) Response of superoxide dismutase, catalase, and ATPase activity in bacteria exposed to acetamiprid. Biomed Environ Sci 19: 309–314. 17044650

[20] Lee C, Kim I, Lee J, Lee KL, Min B, Park C (2010) Transcriptional activation of the aldehyde reductase YqhD by YqhC and its implication in glyoxal metabolism of Escherichia coli K-12. J Bacteriol 192: 4205–4214. 20543070 10.1128/JB.01127-09 PMC2916428

[21] Djaman O, Outten W, Imlay JA (2004) Repair of oxidized iron–sulfur clusters in *Escherichia coli* . J Biol Chem 279: 44590–44599. 15308657 10.1074/jbc.M406487200

[22] Italiani VC, Zuleta LF, Marques MV (2002) The transcription termination factor Rho is required for oxidative stress survival in Caulobacter crescentus. Mol Microbiol 44: 181–94. 11967078 10.1046/j.1365-2958.2002.02865.x

[23] Kuczynska-Wisnik D, Matuszewska E, Laskowska E (2010) *Escherichia coli* heat-shock proteins IbpA and IbpB affect biofilm formation by influencing the level of extracellular indole. Microbiology 156 ((1)) 148–157. 19797360 10.1099/mic.0.032334-0

[24] Caldas TD, Yaagoubi AE, Richarme G (1998) Chaperone properties of bacterial elongation factor EF-Tu. Journal of Biological Chemistry 273: 11478–11482. 9565560 10.1074/jbc.273.19.11478

[25] Susin MF, Baldini RL, Gueiros-Filho F, Gomes SL (2006) GroES/GroEL and DnaK/DnaJ have distinct roles in stress responses and during cell cycle progression in *Caulobacter crescentus* . J Bacteriol 188: 8044–8053. 16980445 10.1128/JB.00824-06 PMC1698207

[26] St Denis TG, Huang L, Dai T, Hamblin MR (2011) Analysis of the bacterial heat shock response to photodynamic therapy-mediated oxidative stress. Photochem Photobiol 87 ((3)) 707–713. 21261628 10.1111/j.1751-1097.2011.00902.x PMC3082629

[27] Boudvillain M, Figueroa-Bossi N, Bossi L (2013) Terminator still moving forward: expanding roles for Rho factor. Curr Opin Microbiol 16: 118–124. 23347833 10.1016/j.mib.2012.12.003

[28] Gao Y, Peng X, Zhang J, Zhao J, Li Y, et al (2013) Cellular response of E. coli upon Hg_2+_ exposure - a case study of advanced nuclear analytical approach to metalloproteomics. Metallomics 5: 913–919. 23771180 10.1039/c3mt20279h

[29] Kobayashi R, Suzuki T, Yoshida M (2007) *Escherichia coli* phage-shock protein A (PspA) binds to membrane phospholipids and repairs proton leakage of the damaged membranes. Mol Microbiol 66: 100–109. 17725563 10.1111/j.1365-2958.2007.05893.x

[30] Wang H, Oster G (1998) Energy transduction in the F1 motor of ATP synthase. Nature 396: 279–282. 9834036 10.1038/24409

[31] Lohmeier-Vogel E, Ung S, Turner R (2004) In vivo 31 P nuclear magnetic resonance investigation of tellurite toxicity in Escherichia coli. Appl Environ Microbiol 70: 7342–7347. 15574934 10.1128/AEM.70.12.7342-7347.2004 PMC535159

[32] Burian J, Nguyen T, Klucar L, Guller L, Lloyd-Jones G, et al (1998) In vivo and in vitro cloning and phenotype characterization of tellurite resistance determinant conferred by plasmid pTE53 of a clinical isolate of Escherichia coli. Folia Microbiol 43: 589–599. 10069007 10.1007/BF02816374

[33] Candiano G, Bruschi M, Musante L, Santucci L, Ghiggeri M (2004) Blue silver: A very sensitive colloidal Coomassie G-250 staining for proteome analysis. Electrophoresis 25: 1327–1333. 15174055 10.1002/elps.200305844

[34] Sunyer B, Diao WF, Kang SU, An G, Boddul S, et al (2008) Cognitive enhancement by SGS742 in OF1 mice is linked to specific hippocampal protein expression. J Proteome Res 7: 5237–5253. 19367722 10.1021/pr800594b

[35] Schellhorn HE, Stones VL (1992) Regulation of *katF* and *katE* in *Escherichia coli* K-12 by weak acids. J Bacteriol 174: 4769–4776. 1385595 10.1128/jb.174.14.4769-4776.1992 PMC206274

[36] Beers RF, Sizer J, Sizer IW (1952) A spectrophotometric method for measuring the breakdown of hydrogen peroxide by catalase. J Biol Chem 195: 133. 14938361

