# Supplementary figures and images for: Proteomic Differences between Tellurite-Sensitive and Tellurite–Resistant E.coli
Source: PLoS One. 2013 Nov 11;8(11):e78010. doi: 10.1371/journal.pone.0078010 (PMC3823874; doi:10.1371/journal.pone.0078010)

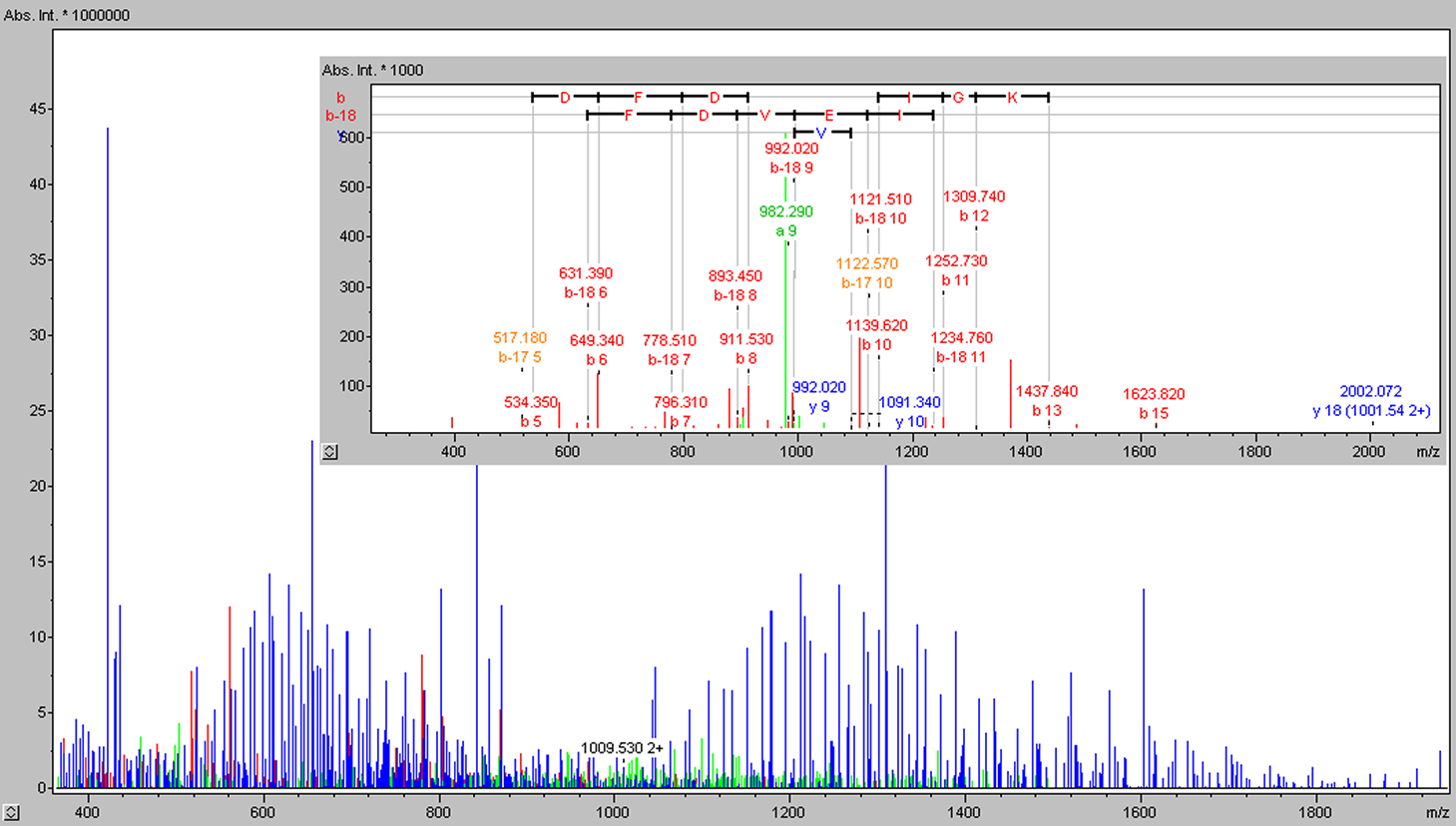

Supplement: Figure S1 — Representative CID MS/MS spectra of tryptic peptide KLVFAVTIYDAEARKQNFGMVSNDFMR of TerB protein. (TIF) [file pone.0078010.s001.tif]

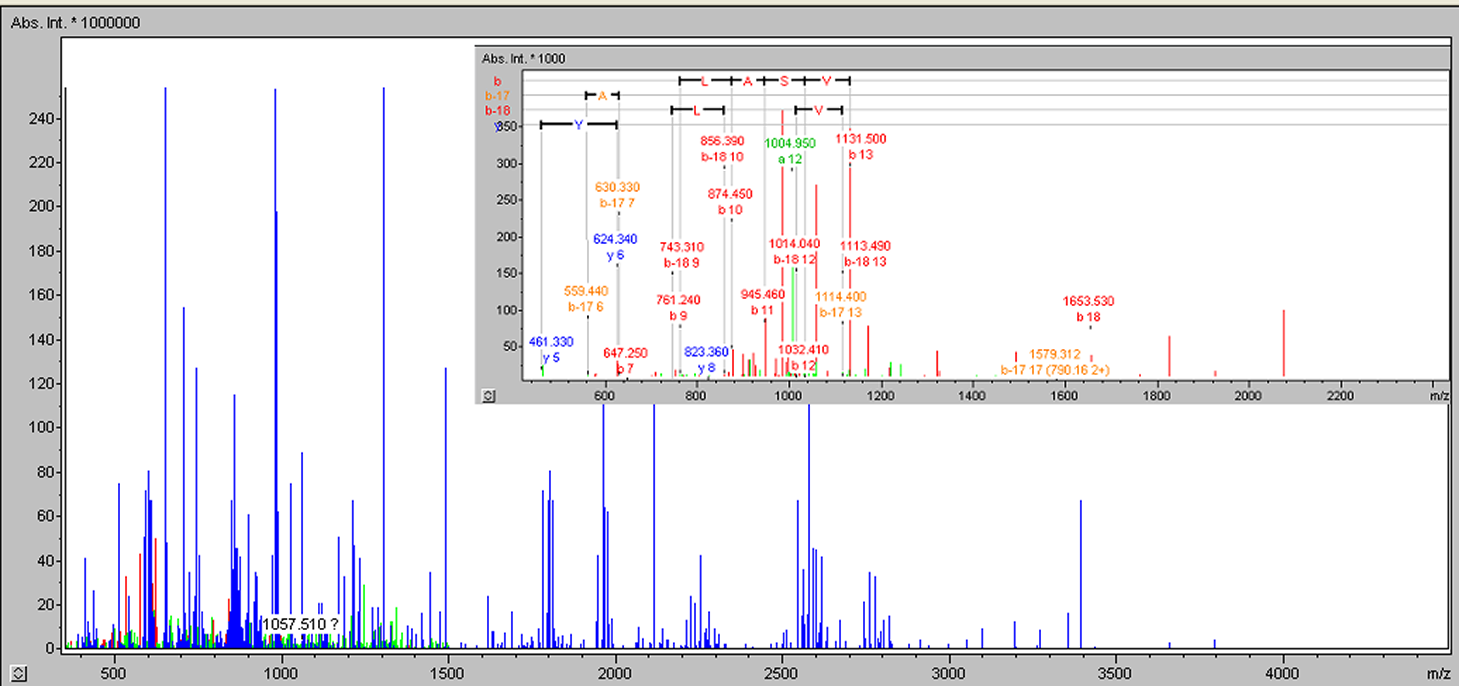

Supplement: Figure S2 — Representative CID MS/MS spectra of tryptic peptide FAGFLYIHHGAELASVFVTGYALEK of TerC protein. (TIF) [file pone.0078010.s002.tif]

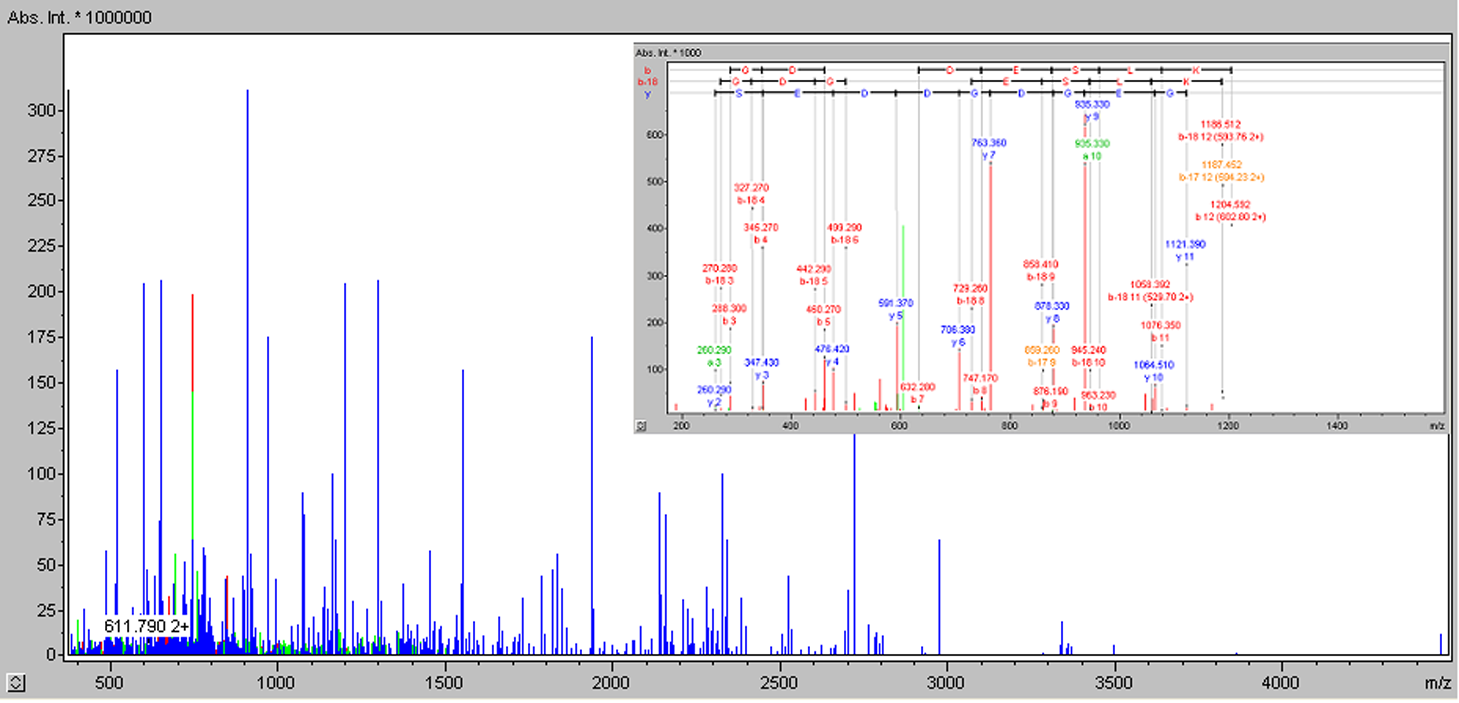

Supplement: Figure S3 — Representative CID MS/MS spectra of tryptic peptide TGEGDGDDESLK of TerD protein. (TIF) [file pone.0078010.s003.tif]

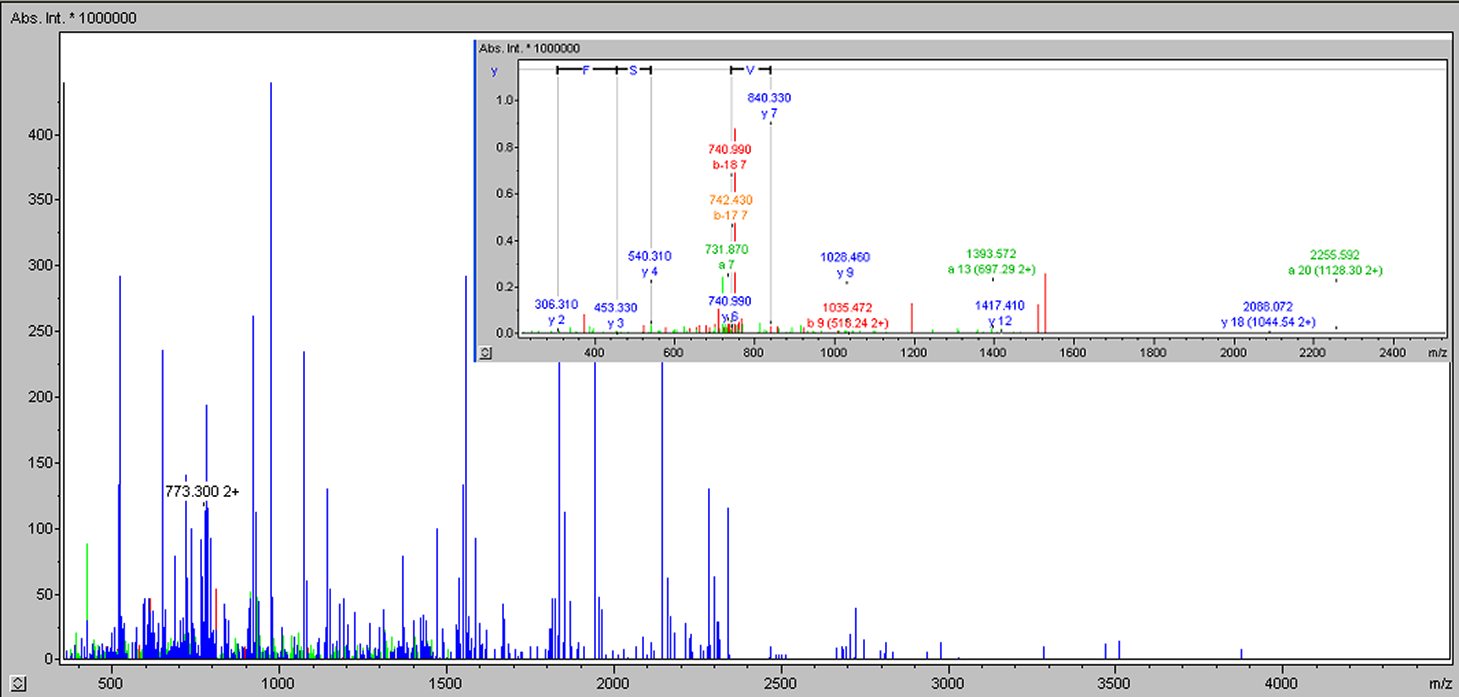

Supplement: Figure S4 — Representative CID MS/MS spectra of tryptic peptide KQNFGMVSNSFMR of TerE protein. (TIF) [file pone.0078010.s004.tif]
